# Vertebral Artery Dissection and Lateral Medullary Syndrome Following a High-Intensity CrossFit Workout

**DOI:** 10.7759/cureus.97420

**Published:** 2025-11-21

**Authors:** May Oo Cho, Thet Paing Oo, Kaung Htet Kyaw, Thin Thin Swe, Chit Aung Hmu

**Affiliations:** 1 Stroke Medicine, Norfolk and Norwich University Hospitals NHS Foundation Trust, Norwich, GBR; 2 Trauma and Orthopaedics, University Hospitals Dorset, Poole, GBR; 3 Stroke Medicine, East and North Hertfordshire NHS Teaching Trust, Stevenage, GBR; 4 General Surgery, Chelsea and Westminster Hospital NHS Foundation Trust, London, GBR

**Keywords:** acute ischaemic stroke, delay presentation, high-intensity crossfit workout, lateral medullary syndrome (wallenberg syndrome), migraine like symptom, posterior circulation stroke, vertebral artery dissection

## Abstract

Cervical artery dissection (CAD), including carotid and vertebral artery dissection (VAD), is an uncommon but important cause of ischemic stroke in young adults. We report the case of a 32-year-old previously healthy woman who developed lateral medullary (Wallenberg) syndrome secondary to a right VAD following a high-intensity CrossFit session involving heavy lifting. She initially presented with right-sided headache and dizziness, which were misattributed to migraine, and over the following days developed dysphagia, right facial hypoesthesia, Horner’s syndrome, and contralateral sensory loss. Computed tomography angiography (CTA) confirmed right VAD, and brain magnetic resonance imaging (MRI) demonstrated a lateral medullary infarction. The patient was treated with dual antiplatelet therapy (DAPT) and underwent multidisciplinary neurorehabilitation, achieving complete functional recovery (modified Rankin Scale (mRS) score = 0). This case underscores the importance of considering VAD in young patients presenting with severe or atypical headaches after strenuous activity, even when initial neurological examination and imaging appear normal, as early recognition and treatment are crucial to prevent disabling stroke outcomes.

## Introduction

Cervical artery dissection (CAD), including carotid and vertebral artery dissection (VAD), represents an uncommon yet significant cause of ischemic stroke in young and middle-aged adults, accounting for up to 20% of cases in this population [1]. It results from a tear in the arterial wall, leading to intramural hematoma formation and subsequent stenosis, occlusion, or embolic infarction. While some dissections occur spontaneously, many are precipitated by minor neck trauma or vigorous physical activity, such as weightlifting or CrossFit training, which impose mechanical and hemodynamic stress on the cervical arteries [2,3]. The initial symptoms, such as headache, neck pain, and dizziness, are often nonspecific and easily mistaken for benign conditions like migraine, contributing to diagnostic delays. Here, we report a case of VAD resulting in lateral medullary (Wallenberg) syndrome following a high-intensity CrossFit workout, highlighting the importance of clinical suspicion in young patients presenting with atypical headaches after strenuous exercise.

## Case presentation

A 32-year-old generally fit and active woman developed a right-sided headache and dizziness following her routine CrossFit session involving heavy weightlifting. Unlike her usual migraine episodes, the pain persisted despite analgesics for two days, prompting a visit to her general practitioner (GP). Clinical examination was unremarkable, and a diagnosis of migraine was made. 

Later the same day, after her GP visit, she suddenly developed new symptoms, including choking on water, shooting pain on the right side of her face, and an unsteady gait while at a walk-in center. This prompted a call to the paramedics, who transported her to the hospital. However, given her young age, absence of classic stroke signs (such as slurred speech, facial droop, or limb weakness), and atypical presentation, referral to the stroke team was delayed.

**Figure 1:**
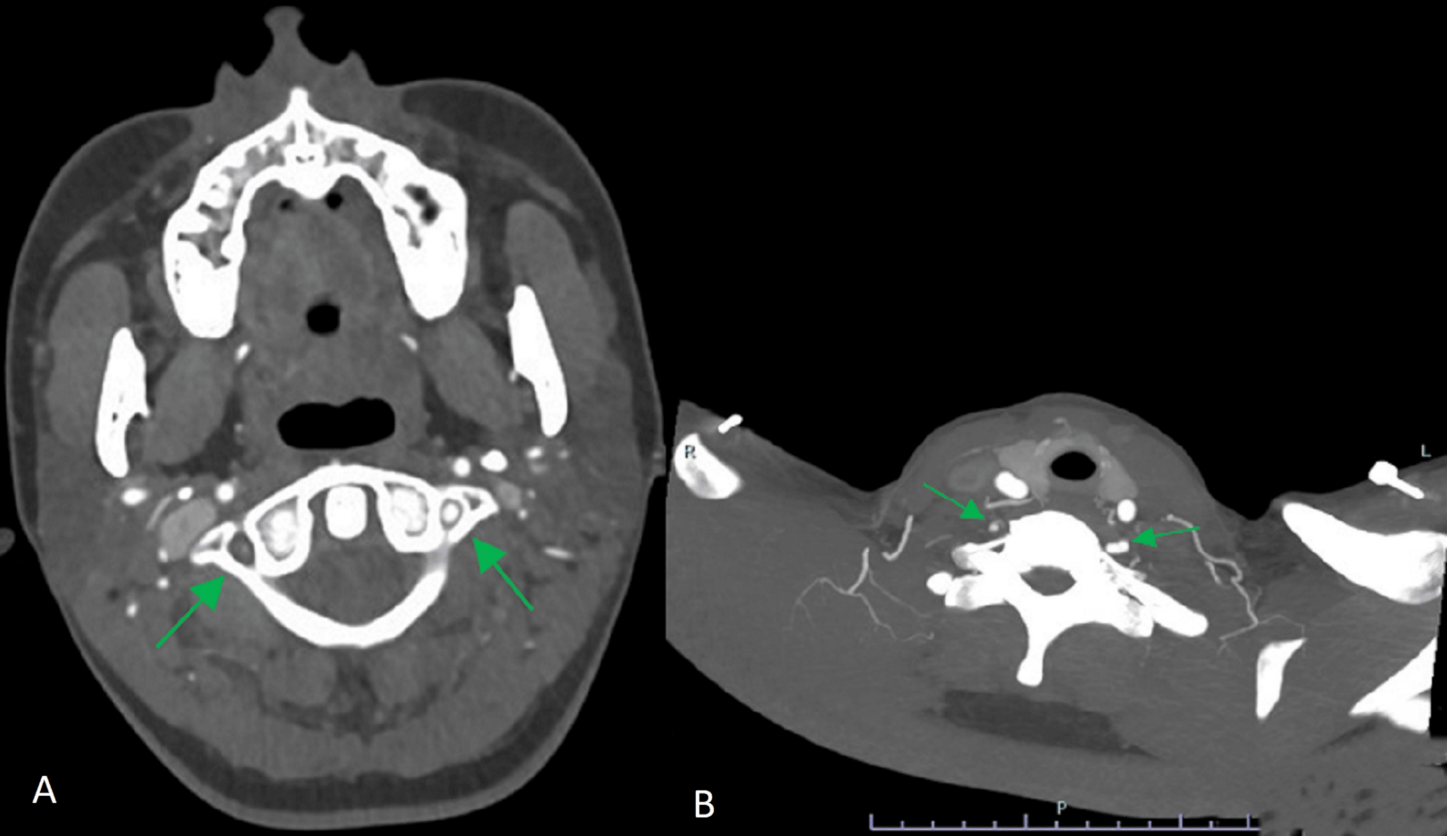
(A) and (B) CTA images showing loss of contrast flow within the right vertebral artery, with evidence of an intimal flap suggesting VAD. The left vertebral artery demonstrates normal opacification and caliber. CTA, computed tomography angiography; VAD, vertebral artery dissection.

On assessment, she exhibited right-sided facial hypoesthesia, dysphagia, and Horner’s syndrome (miotic, fixed right pupil, and ipsilateral ptosis), with a National Institutes of Health Stroke Scale (NIHSS) score of 3 [4]. Non-contrast computed tomography (CT) of the brain was normal, but computed tomography angiography (CTA) revealed a right VAD, as shown in Figure 1. She was diagnosed with posterior circulation stroke (POCS) secondary to VAD, likely triggered by high-intensity exercise. As symptom onset was beyond 48 hours, intravenous thrombolysis and mechanical thrombectomy were not indicated. Dual antiplatelet therapy (DAPT) was initiated.

**Figure 2:**
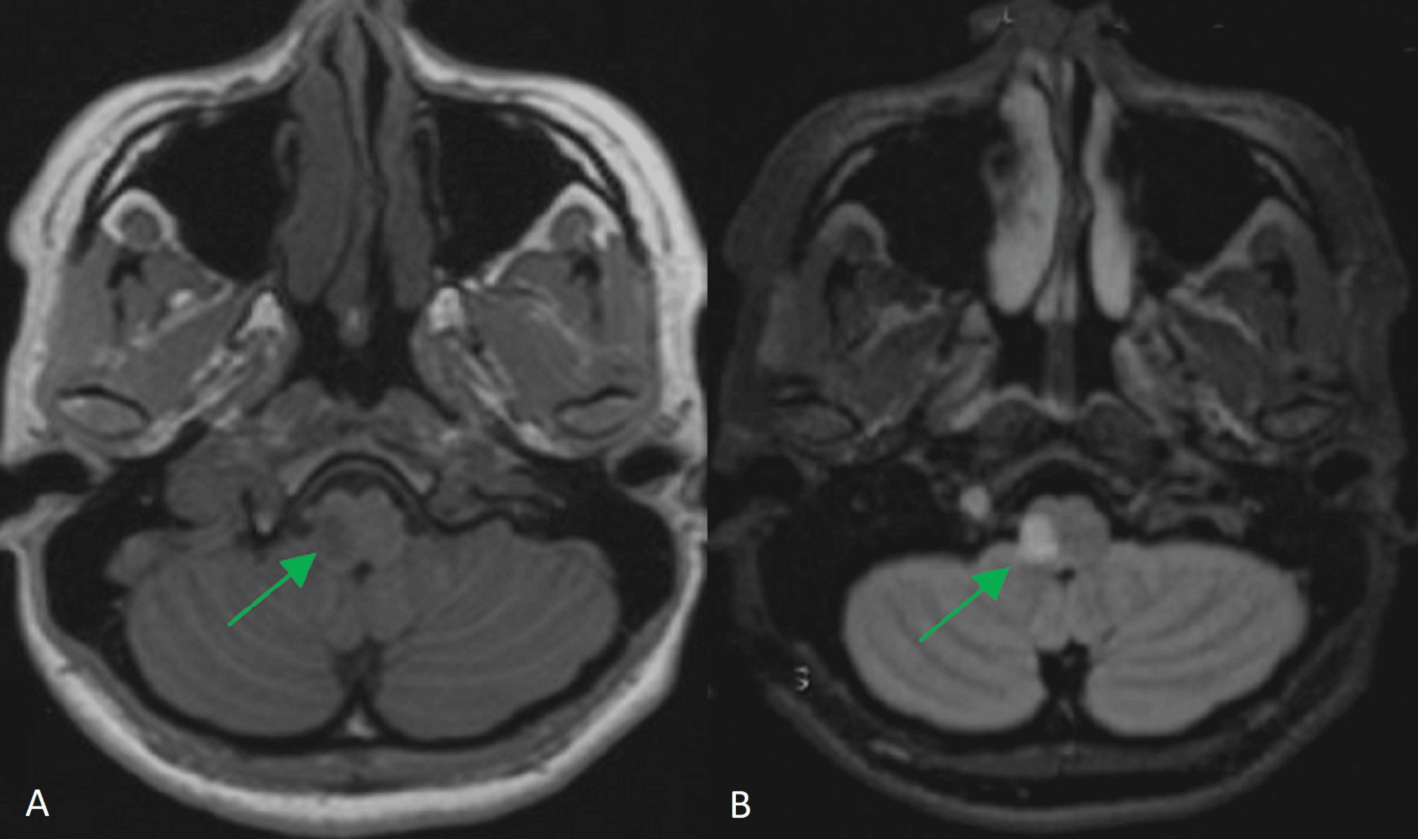
(A) T1-weighted and (B) T2-weighted axial MRI sequences showing a focal infarct in the right lateral medulla oblongata (ventrolateral region). MRI, magnetic resonance imaging.

**Figure 3:**
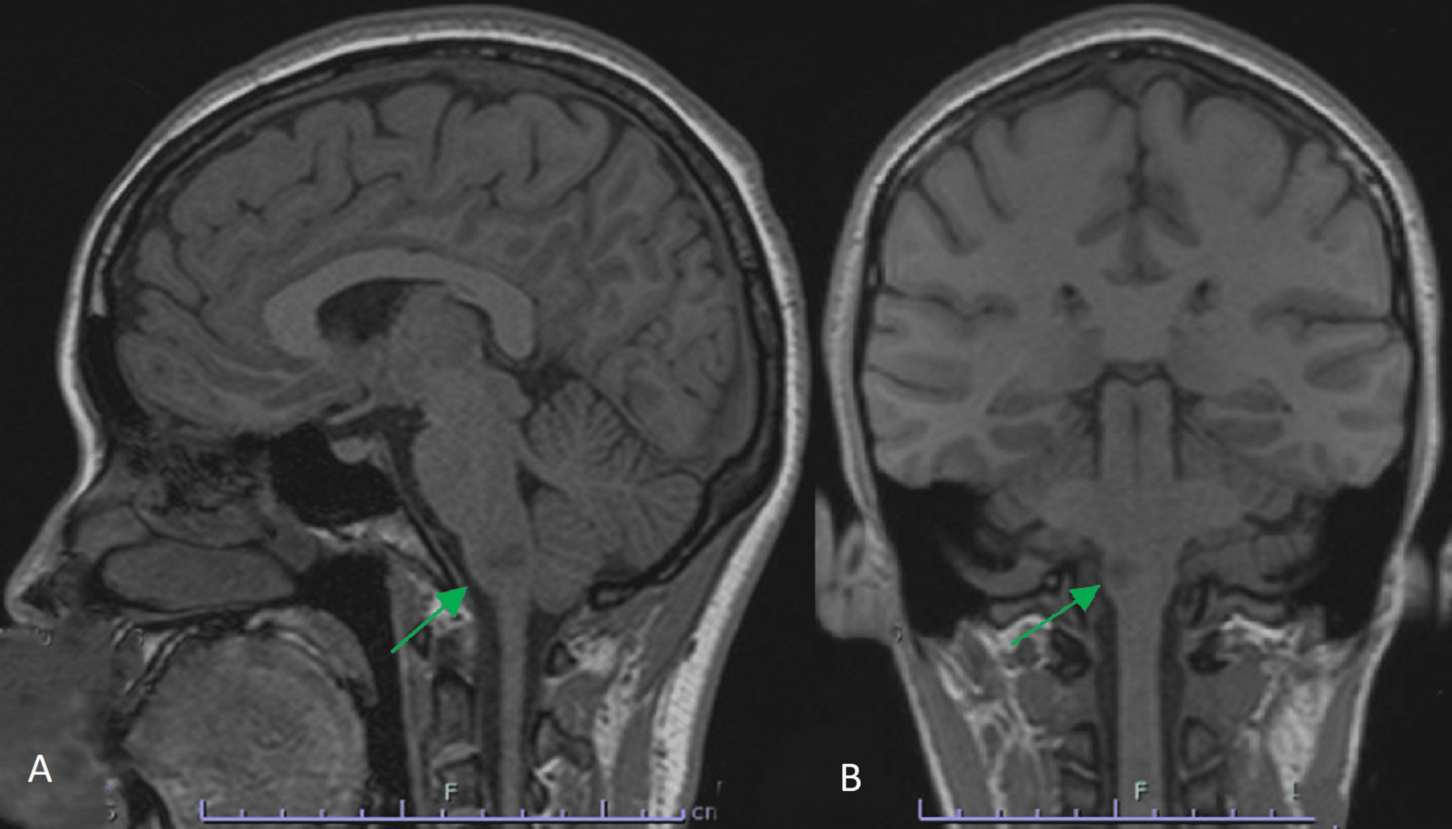
(A) Sagittal and (B) coronal MRI views confirming the extent of the lateral medullary infarction. MRI, magnetic resonance imaging.

Over the following days of her admission, she developed contralateral loss of pain and temperature sensation and hoarseness, consistent with evolving Wallenberg syndrome. Magnetic resonance imaging (MRI) of the brain and magnetic resonance angiography (MRA) confirmed a right lateral medullary infarction secondary to a right VAD, as shown in Figure 2 and Figure 3. Screening for connective tissue disorders was unremarkable, and there was no family history of vascular or connective tissue disease. She underwent multidisciplinary neurorehabilitation, including speech and language therapy for dysphagia and physiotherapy for ataxia. Her symptoms gradually improved, and she was discharged with only a mild residual left-sided sensory deficit and a modified Rankin Scale (mRS) score of 0.

## Discussion

This case illustrates VAD as an important differential diagnosis in young patients presenting with atypical headaches after high-intensity exercise involving abrupt neck movements [1]. Activities such as CrossFit, yoga, and contact sports have been linked to CAD through mechanisms of hyperextension, rotation, or direct trauma to the cervical arteries [1,5]. Although spontaneous dissections occur without identifiable trauma, vigorous exercise can serve as a mechanical trigger in predisposed individuals, even in the absence of underlying connective tissue disorders [5].

The pathophysiology of ischemic stroke in VAD involves two primary mechanisms:

Thromboembolism: An intimal tear allows blood to enter the vessel wall, forming an intramural hematoma and promoting thrombus formation. Embolic fragments may migrate distally to occlude intracranial arteries, resulting in infarction [6].

Hemodynamic compromise: Expansion of the intramural hematoma can cause luminal narrowing or complete occlusion, leading to reduced perfusion in the vertebrobasilar system and subsequent ischemia.

Both mechanisms may coexist, and in many cases of lateral medullary syndrome, the predominant cause is artery-to-artery embolism from the dissected vertebral segment [7].

Early recognition of dissection-related POCS is essential, as initial symptoms can mimic benign conditions like migraine. Delays in diagnosis may preclude timely thrombolysis. Although both CTA and MRA are used, there is no established gold standard, and combining modalities improves diagnostic accuracy [8].

Management typically involves antithrombotic therapy, but evidence remains limited regarding whether antiplatelets or anticoagulants are superior [8]. Thrombolysis is generally considered safe in ischemic stroke due to arterial dissection when administered within the therapeutic window; however, in intracranial dissections, there may be increased risk of hemorrhagic transformation or early neurological deterioration [9,10].

## Conclusions

In young patients presenting with severe or atypical headaches, especially following strenuous physical activity, arterial dissection should be considered once intracranial hemorrhage is excluded. Prompt vascular imaging and early specialist assessment are essential to prevent diagnostic delays and optimize neurological outcomes.
